# Correlation Tests of Ultrasonic Wave and Mechanical Parameters of Spot-Welded Joints

**DOI:** 10.3390/ma15051701

**Published:** 2022-02-24

**Authors:** Dariusz Ulbrich, Marta Kańczurzewska

**Affiliations:** 1 Faculty of Transport and Civil Engineering, Institute of Machines and Motor Vehicles, Poznan University of Technology, 60-965 Poznan, Poland; 2 Faculty of Control, Robotics and Electrical Engineering, Poznan University of Technology, 60-965 Poznan, Poland; marta.kanczurzewska@put.poznan.pl

**Keywords:** ultrasound, spot welding, joint strength, multiple regression, logistic regression, decision tree, random forest, machine learning

## Abstract

Resistance spot welding as the basic method of joining car body elements has been used in the automotive industry for many years. For these connections, it is required to obtain the appropriate diameter of the weld nugget, which results in a high strength and durability of the connection during vehicle operation. The article presents the methodology of testing spot-welded joints using both destructive methods: shearing test of the spot weld and the ultrasonic method. The main goals of the performed tests are (1) to determine the correlation between the mechanical strength of a joint, measured in kN, and the selected parameters of the ultrasonic longitudinal wave with a frequency of 20 MHz propagating in the area of the spot weld and (2) to build and verify the predictive models of the weld nugget quality. The correlation of these parameters allows assessing the strength of the connection with the use of a non-destructive test method. On the basis of the performed analyses, it was determined that there is a strongly positive correlation between the number of reverse echoes and the force necessary to destroy the spot weld (0.41) and the diameter of the weld nugget (0.50). A strong negative correlation was also obtained between the number of echoes and the strength (−0.69) and diameter of the weld nugget (−0.72).

## 1. Introduction

The spot welding technology is the basic process of joining car body elements [1,2]. Despite the technological progress and the introduction of exorbitant quality control standards in factories, there are faulty connections that may weaken the stiffness of the car body structure. This is important for the operation of the vehicle. Farrahi et al. proved the influence of the road type and vehicle speed on cracks and damage to the vehicle body, including spot-welded joints, during the operation period [3]. All kinds of cracks in the vehicle body also cause degradation of the vehicle crashworthiness [4,5]. The place of initiation of such a crack may be a defective welded joint [6]. Therefore, much research is being conducted on the improvement of the spot-welding process of materials used in the automotive industry [7,8,9]. Kishore et al. [10] compared the processes of welding for steel with and without a zinc coating and found that during the welding process, the coating on the surface of the steel reduces the diameter of the weld nugget. Soomro et al. [11] proposed the use of double pulse resistance spot welding as a postweld heat treatment method that improves the mechanical properties of the spot weld. On the basis of the research, it was found that for galvanized dual phase (DP590) steel, the most important parameter influencing the mechanical performance of the spot weld is the welding current, then the welding time and the cooling time (for double pulse resistance spot welding). In the case of welding very thin steel sheets (less than 0.6 mm), there are significant problems related to heat transfer to the electrode surface from the welded joint zone, which affects the durability of the electrodes (reduces the life of the electrodes). Therefore, there are attempts to reduce this phenomenon by adding additional strips between the welded material and the electrodes [12]. This makes it possible to limit the temperature on the electrode surface, to initiate the production of the weld joint earlier, as well as to obtain a larger diameter of the weld nugget.

Other studies [13] show that for a cyclic fatigue performance with low load, it is better to make welds with a smaller nugget diameter, because in the case of a larger diameter of the weld nugget, crack growth is accelerated.

Feujofack Kemda et al. [14] developed surface models that combine the hardness of the weld and its diameter with the basic parameters of the welding process. On the basis of these models, it was found that the welding current is the dominant parameter and should be maximized in order to obtain strong spot welds. Other research has focused on developing a spot weld model based on the electrical, thermal, mechanical, and metallurgical parameters, which are essential during the spot welding process [15]. The advantage of the developed model is the ability to determine the parameters of the spot welding process, which will ensure the optimal fusion zone limiting the cracking of the liquid metal around the spot welds. A correctly made connection provides the required stiffness and durability of the entire structure of the vehicle body, which undoubtedly improves the safety of road vehicles. A separate group consists of modeling works in the field of determining the shape and diameter of the weld nugget and predicting the durability of a spot-welded joint on the basis of the finite element method [16,17,18].

Nevertheless, the most important from the point of view of the car body manufacturer are the methods and technologies that allow assessing the quality of the welded joint. In industrial practice, both destructive (e.g., peel test) and non-destructive methods–ultrasonic inspections [19] are used. Destructive methods can be used randomly; they do not allow for the control of every joint produced, due to the nature of the test destruction of the joint. Moreover, these methods are time consuming and require appropriate laboratory facilities. Other methods of non-invasive evaluation of the quality of spot-welded joints include digital shearography [20], infrared thermography [21], neutron radiography [22], visual assessment [23], as well as the penetration and eddy current method [24]. The neutron radiography method allows for the visualization of the weld nugget, which is also possible if the ultrasonic wave impulse is properly processed [25].

Ultrasonic tests of spot-welded joints come down to the assessment of pulses of ultrasonic longitudinal wave propagating in the area of the joint [26,27]. From the point of view of the joint assessment, important factors include the number of reverse pulses from the bottom of the joint (also the amplitude of subsequent echoes), the number of reverse pulses from the area of the sheet–intermediate echo, and the thickness of the weld at the joint, which is measured with the ultrasonic method [28].

In recent years, significant progress has been made in the study of ultrasonic spot-welded joints. Amiri et al. [29] investigated by neutral network the relationship between the results of ultrasonic testing with tensile strength and fatigue life of three-sheet spot-welded joints. Based on the research results, a new methodology for the assessment of the static strength and fatigue life of three-sheet welded joints based on the ultrasonic method was proposed. Qiuyue et al. [30] proposed a method for the detection and evaluation of the amount of porosity based on the wavelet packet analysis, which is more accurate (accuracy limited to 7%) than the so far used ultrasonic wave pulses analysis. Additionally, automatic ultrasound control was used [31], as well as online evaluation of the connection during its production [32]. Nevertheless, in many factories producing car bodies for motor vehicles, standard ultrasonic inspection is performed by the operator. The evaluation of the welded joint using the ultrasonic method allows to determine its quality and occurrence of defects. Nevertheless, it is important from the point of view of the car body strength to determine the relationship between the parameters of the ultrasonic longitudinal wave and the mechanical strength MPa of the welded joint.

The few attempts to determine the correlation between ultrasonic wave parameters and shear force [33] can be summarized as follows: when the attenuation coefficient increases, the tension–shear strength of the spot weld increases as well. However, the attenuation coefficient is not determined with standard ultrasonic control performed in factories. Therefore, the authors focused on other parameters of the ultrasonic wave.

The main purpose of the research presented in this article was to determine the correlation between the parameters of the ultrasonic longitudinal wave propagating in the area of the spot-welded joint and its mechanical strength expressed in kN as well as the diameter of the weld nugget. The research articles described above deal with the subject of ultrasonic research of spot-welded joints. These tests have been carried out in factories that produce vehicles for many years. Nevertheless, there is no reference of the ultrasonic wave parameters to the mechanical parameters of the welded joint e.g., expressed in kN. It has also not been verified what the thickness of the spot weld should be in the place of joining the sheets, which allows for the production of a strong joint with adequate shear strength. Particularly, there are no correlations determined for the connections of thin car body sheets. The basic parameters of the ultrasound wave, tested in the conditions of a factory producing vehicles or a body shop, allow only for a non-destructive estimation of the quality of the connection.

It is important from the point of view of the passenger safety of the vehicle, especially one that has undergone sheet metal repair, to check the strength of spot-welded joints. Therefore, it should be stated that the main novelty contained in the article, in relation to the existing test results, is the determination of the value ranges of the destructive force and the weld nugget in relation to the parameters of the ultrasonic wave. Determining the ranges of values of the ultrasonic wave parameters, such as the number of reverse echoes, the thickness of the weld at the joint in relation to the destroying force of the joint, and the weld nugget diameter will be the first stage in the development of the method of estimating the strength of the spot weld using the ultrasonic method.

## 2. Materials and Methods

### 2.1. Resistant Spot Welding Background

Resistance spot welding is a process in which a connection can be obtained by heating the contact areas of the joined elements by the electric current flowing through them and plastic deformation of this area due to the appropriate pressure force. The heating of the objects’ area during resistance spot welding takes place through the release of thermal energy on the electrical resistances of the welding circuit when an electric current flows through them. The Joule–Lenz formula determines the amount of heat energy released in the area of the elements located between the electrodes supplying the welding current at individual resistances and is as follows:

(1) $$Q=\int_{0}^{t_{z}}J^{2}\left(t\right)R\left(t\right)\mathrm{dt}$$

where J(t) is the welding current intensity, R(t) is the total electrical resistance of the welding point, and t_z_ is the welding time in seconds.

Based on the research results [34,35,36], it can be concluded that the welding current and welding time are two main factors that affect the diameter of the weld nugget. These two factors are included in Equation (1), and the quality of the joint depends on them (this quality is understood as the diameter of the weld nugget, which affects the mechanical strength of the joint). Changes in these two parameters of the welding process cause the possibility of defects in the joints, which lead to the weakening of the strength not only of the joint itself but the entire structure. However, the shear strength of the joint can also depend on the steel sheet thickness and the base-metal strength.

The next main factor that influences the spot welding process (mainly residual stress and weld nugget) is distribution and changes in plastic strain. It is important to determine and verify thermal changes during the spot welding, which causes the plastic deformation. Wang et al. [37] proposed thermal analysis by the Fourier law:

(2) $$\rho c\frac{\partial T}{\partial t}=\frac{\partial }{\partial x}\left(k_{x}\frac{\partial T}{\partial x}\right)+\frac{\partial }{\partial y}\left(k_{y}\frac{\partial T}{\partial y}\right)+\frac{\partial }{\partial z}\left(k_{z}\frac{\partial T}{\partial z}\right)+q_{v}$$

where ρ is the density of the material, c is the specific heat capacity of the material, T is the temperature, t is the time, k_x_, k_y_, and k_z_ are thermal conductivity for the directions (for isotropic material k_x_ = k_y_ = k_z_ = k, and q_v_ is the rate of internal heat generation.

Plastic deformation is also caused by the pressing force of the electrodes during the welding process. The above-described factors have a significant impact on the appearance of defects in the welded joints. Therefore, in the research presented in the next part of the manuscript, one of the parameters of the welding process (welding current) was selected, which was variable and determined the quality of the joint.

### 2.2. Samples

**Figure 1:**
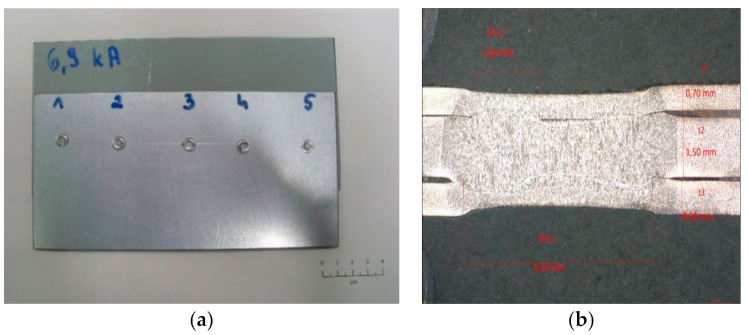
Samples used during the test: (**a**) five spot welds on one sample, (**b**) cross-section of spot weld.

The tests were carried out on samples made of 0.8 and 1.2 mm thick sheets. The 0.8 mm thick sheet was electrolytically galvanized on both sides, while the thicker sheet used during the tests was hot-dip galvanized on both sides. Car body sheets from the production line of car bodies were used in the research (samples were cut from body parts). The authors did not check the surface parameters, because identically prepared sheets are used in the construction of vehicles. For the selected parameters of the welding process, 5 welded joints were made, and then, the sheets were cut to obtain five separate samples with dimensions of approximately 40 × 100 mm with a weld nugget in the center of the sample. The view of samples used in the tests is shown in Figure 1a, and the cross-section of an additional spot weld (that was not destroyed in shear test) is shown in Figure 1b.

The parameters of the welding process, such as the pressure of the electrodes (spot-welding force), the welding time, and the number of welding pulses were determined on the basis of the data of vehicle manufacturers. In order to obtain spot welds of different quality, it was necessary to select one parameter that would be changed—the welding current. The welding current values were selected based on the experience of the authors of the work in order to diversify the nugget diameter of the spot welds. The welding current varied from 5.5 to 7.7 kA with a step of 0.2 kA. Additionally, spot welds at welding currents of 8.0 and 8.5 kA were made. The variable welding current allows making connections with different diameters of the weld nugget, which correlates with its strength. Copper electrodes with a diameter of 16 mm were used for spot welding, and the electrode face was 4.5 mm. The detailed parameters of the process of joining sheets and producing a spot weld on the samples were as follows:

- Spot-welding force 2.5 kN;
- Spot-welding current 5.5 ÷ 8.5 kA;
- Spot-welding time 0.28 s;
- Number of welding pulses 2;
- Time before spot welding (only welding force) 0.8 s;
- Time after spot welding (cooling) 0.14 s;
- Ignition angle 90°.

For each welding current, 5 samples were made, while maintaining the other parameters of the process. The production of several welded joints side by side on a piece of steel sheet requires the maintenance of appropriate distances between the individual spot welds. This is due to the shunting effect, i.e., the partial flow of current through the closest weld already produced instead of the actual place where the connection is made. The phenomenon of shunting the current may cause the successive connections produced to be of poor quality and have too small a spot weld nugget. In order to avoid this phenomenon (for the selected combination of sheets), the arrangement of spot welds was used in accordance with the body repair manual of one of the car manufacturers.

The joints were produced according to the above parameters, and the shear test was performed after ultrasonic testing to determine the stresses destroying the spot-welding joint.

### 2.3. Ultrasonic Testing

**Figure 2:**
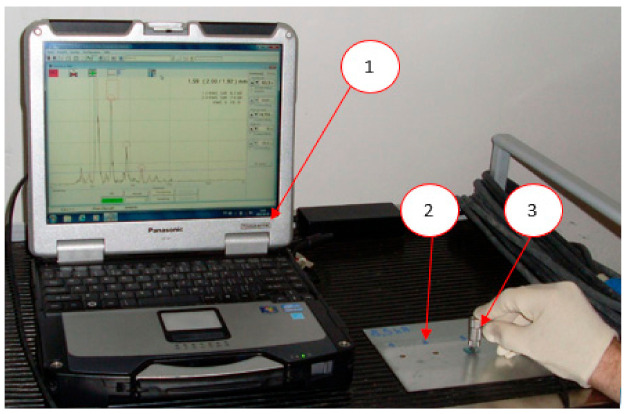
Examination of the welded joint using the ultrasonic method; 1—ultrasonic flaw detector, 2—sample with spot welds, 3—ultrasonic probe.

The ultrasonic tests were carried out with the use of the USLT 2000 ultrasonic flaw detector (Krautkramer GE, Boston, MA, USA) and the 20 MHz probe (Krautkramer GE, Boston, MA, USA) with water delay. The diameter of the head transducer, amounting to 4.0 mm, was selected on the basis of the sheet thickness of 0.8 mm and the formula for the transducer diameter. The view of the flaw detector during the measurements is shown in Figure 2.

Each spot weld was tested with an ultrasonic longitudinal wave 7 times. The number of measurements of one spot weld was determined on the basis of previously performed 40 measurements on one spot weld and the determination of the coefficient of variation. During the measurements, the number of reverse echoes, the number of intermediate echoes, and the RWS parameter (the thickness of the sheets after the connection) were recorded.

During ultrasonic testing, there are certain factors that affect the correctness of measurements and the obtained results. In the case of testing thin car body sheets, it is important to use high-frequency ultrasonic transducers (e.g., 15, 20 MHz). It is necessary to generate an ultrasonic wave in the tested material (spot weld). In addition, the transducer itself is located at a distance from the test site (water delay chamber) in order to avoid interference of the ultrasonic wave signal affecting the results. If the high-frequency transducer was placed directly at the spot weld, the test would be conducted in the near field, where there are significant fluctuations in sound pressure that affect the measurement result. Impressions on the electrodes are a characteristic feature of the spot weld. That is why ultrasonic testing uses heads with a rubber membrane that adjusts to the shape of the stamp. This allows the ultrasonic wave beam to be introduced perpendicular into the area of the tested weld. Any deviations from this rule result in reflection of the ultrasonic wave from the bottom of the connection at an angle and the inability to obtain a diagnostic signal and information about the connection quality. Sometimes, a stamp of the electrodes causes sharp edges that damage the rubber membrane (water comes out of the chamber, which also contains the transducer). This makes it necessary to replace the membrane with a new one. Additionally, the membrane and the coupling gel, which is applied to the surface at the spot weld, eliminate the influence of surface defects (e.g., scratches) on the ultrasonic signal from the joint. Moreover, it should be stated that the difficulties in the ultrasonic tests of the entire car body are welds made on sheets of different thickness. The ultrasonic head (diameter of the transducer) is selected on the basis of the thickness of the sheets to be joined. Therefore, in the case of a large number of joints, the transducers should be changed, adapting them to a part of the car body and spot-welded joints.

### 2.4. Mechanical Testing

**Figure 3:**
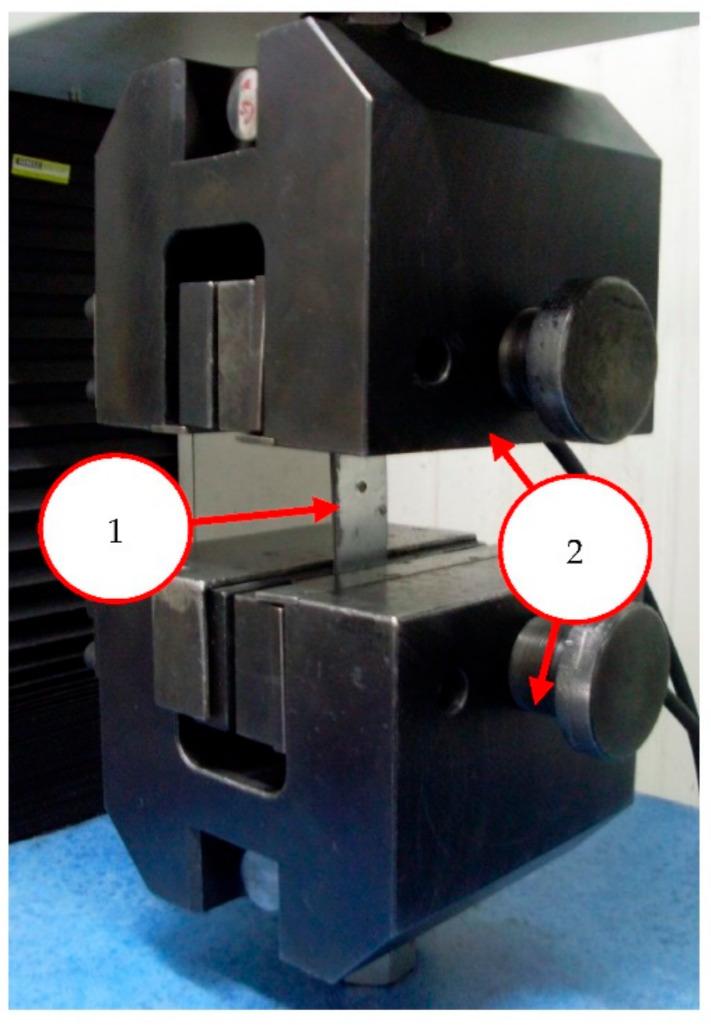
Sample in testing machine jaws; 1—sample, 2—jaws.

Samples with spot-welded joints of different quality, after ultrasonic testing, were shorn on a testing machine (Instron, Norwood, MA, USA). That allowed determining the destroying force of the joint. The speed of movement of the jaws of the testing machine was set at 25.0 mm/min, which corresponds to the values used by one of the world’s biggest manufacturers of motor vehicles in the strength tests of spot-welded joints. The maximum test time was set to 120 s. However, for most of the tested spot-welded joints, the joint was damaged after around 15 s. It was observed by the separation of the sheets in the place where the spot weld had previously formed. The final result of the shear test was the value of the destroying force of the joint and the graphs of the force change depending on the distance traveled by the jaws of the testing machine (in mm). Only the value of the destructive force was used in the analysis of the results. The view of the sample in the jaws of the testing machine is shown in Figure 3.

### 2.5. Statistical Analysis

Statistical analysis was performed using RStudio software (R version 1.2.5001) Rstudio Team (2019). Each of the tested samples before the destructive test was evaluated seven times with the non-destructive ultrasonic method (three parameters were determined: the number of reverse echoes, the number of intermediate echoes, and the RWS parameter). Before the statistical analysis, results of these parameters were averaged.

The main analysis was divided into two stages. The first stage involved a comparative analysis with the division of data into two groups—samples where the quality of the connection was assessed as good and those where the connection was assessed as unacceptable. The division criterion was established on the basis of the 4 mm diameter of the weld nugget (too weak or no connection up to 4 mm, good connection above 4 mm). The second step involved the combined analysis of all 75 samples.

As part of the first stage of the analysis, basic descriptive statistics were determined for both groups: minimum, maximum, median, mean, standard deviation, first quantile, and third quantile. Additionally, the confidence intervals of 95% were determined. All data are illustrated in box-plot charts. Moreover, a comparative analysis of the examined parameters (reverse echoes, intermediate echoes, and RWS) was performed, preceded by the Shapiro–Wilk normality test, in order to select an appropriate statistical test [38]. Based on the results obtained from the Shapiro–Wilk normality test, the *t*-test was used for the comparative analysis of reverse echoes for good and poor (weak) connection. The Wilcoxon test for comparative analysis of good and poor connection between echoes and the RWS parameter was used. The tests were performed at the significance level of *p* = 0.05.

As part of the second stage of the analysis, the basic descriptive statistics for all data and the correlation matrix between all tested parameters were determined—parameters were obtained both from the non-destructive test (reserve echoes, intermediate echoes, RWS) and from the destructive test (destructive force and diameter of the nugget). The analyses were carried out for significance at levels: ‘.’ *p* = 0.1, ‘*’ *p* = 0.05, ‘**’ *p* = 0.01, and ‘***’ *p* = 0.001.

In order to determine the relationship between the ultrasonic parameters and the diameter of the weld nugget, four different predictive models were built, the effectiveness of which was then compared. The multiple regression model, the logistic regression model, the decision tree method, and the random forest were considered. The first two of them quantify the relationship between the predictors and the dependent variable; therefore, they allow for a clear, quantitative determination of the impact of changing the value of individual independent variables on the value of the dependent variable. The next two are black box models, and the relationship between the variables is no longer quantitative. The multiple regression model has been established as the simplest dependency model when the relationship between the dependent variables and the independent variable is linear. Due to the small sample size, a logistic regression model, which is a kind of generalization of multiple regression, was also considered. Due to the great popularity of machine learning models, effectively describing both linear and non-linear relationships between variables, two other models were also considered.

In order to compare the models, the data set consisting of 75 samples was randomly divided into two subsets—the training (to build each of the four models) and the testing (to verify the obtained models on the data on which the models were not built) in a ratio of 3:1. Then, the most accurate model was selected in order to indicate the direction of further research.

Multiple regression is a method that provides simple and effective character models:

(3) $$y=b_{0}+b_{1}\cdot x_{1}+b_{2}\cdot x_{2}+\ldots +b_{n}\cdot x_{n}$$

where $b_{i}$ refers to as beta coefficients and measures the relationship between the predictor and the score.

It is easy to interpret (as the value of the predictor $x_{i}$ increases by one, the value of the dependent variable increases on average by the value of $b_{i}$) and has been successfully used in engineering, computer science, biology, economics, and psychology [39,40,41,42,43,44,45,46,47]. It is critical that the necessary assumptions of multiple regression are met, so these were checked first, and then, the multiple regression analysis was carried out [48,49]. The normality of the model residuals was checked by a Q-Q plot (quantile–quantile plot) and confirmed by the Kolmogorov–Smirnov test. Using the Breusch–Pagan test, the assumption of the homoscedasticity of the model was tested.

The other three methods used in the tests are classification models. It means that the independent variable is a variable with only two values that can be classified as binary—1 and 0, respectively. In this paper, such a variable is the diameter of the weld nugget, in which any diameter that was at least 4 mm was classified as 1 (good joint), and otherwise, it was classified as 0 (weak joint).

Logistic regression is a method that generalizes a linear model for the case where the dependent variable is a variable that can be represented in binary form and is expressed as a probability function called Odds:

(4) $$\mathrm{Odds}=\frac{p}{1-p}=e^{\alpha }\;e^{\beta 1\cdot x_{1}+\beta_{2}\cdot x_{2}+\cdots +\beta_{n}\cdot x_{n}}$$

where α is the logistic regression constant and $\beta_{i}$ is the logistic regression coefficients for the i-th decision variable.

It requires that the predictors are not strongly correlated (which has been verified by the variance of inflation factors), that the observations are independent of each other, and there is a linear relationship between the logit of the result and each predictor variable (which was verified visually by checking the scatter plot between each predictor and the logit values). Logit is a function of the form:

(5) $$\mathrm{logit}\left(p\right)=\log\frac{p}{\left(1-p\right)}$$

where p is the result probability.

The significance of the factors in both models was analyzed at the following levels: ‘*’ *p* = 0.05, ‘**’ *p* = 0.01, and ‘***’ *p* = 0.001.

The last two methods (decision tree and random forest) do not provide answers in the form of regression coefficients, which makes them more difficult to interpret. The decision tree method is a predictive machine learning technique that consists in multiple division of data into subspaces in such a way that the results in the final subspace are as homogeneous as possible. The result of the algorithm’s operation is a decision tree, which is a set of decision rules that allow making a classification decision based on the value of the predictors.

The random forest method, on the other hand, consists in building many different models of decision trees from one set of training data by repeatedly using many initial subsets of data and averaging the models. Each tree is built independently of the others. Unfortunately, it does not provide insight into the forest structure but only the impact of individual variables using the average decrease in the Gini coefficient.

All three classification methods has been also, similarly to multiple regression, successfully used in various fields of science [50,51,52].

In the last stage of the statistical analysis, all the constructed models were verified on the set of testing data, assuming a cut-off point of 50%, where a confusion matrix was first constructed for each model (Table 1).

The explanation of the description contained in Table 1 is as follows: True Positive means the number of good joints correctly classified by the model as a good joints, False Positive means the number of weak joints incorrectly classified by the model as good joints, True Negative means the number of weak joints correctly classified by the model as weak joints, and False Negative means the number of good joints incorrectly classified by the model as weak joints.

Then, 6 parameters were determined based on the confusion matrix:

(6) $$\mathrm{accuracy}=\frac{\mathrm{True}\;\mathrm{Positive}\;+\;\mathrm{True}\;\mathrm{Neative}}{\mathrm{True}\;\mathrm{Positive}\;+\;\mathrm{True}\;\mathrm{Neative}\;+\mathrm{False}\;\mathrm{Positive}\;+\mathrm{False}\;\mathrm{Neative}}$$

which express how often the model correctly predicted the weld nugget quality;

Misclassification error rate

(7) $$\mathrm{MER}=\frac{\mathrm{False}\;\mathrm{Positive}\;+\mathrm{False}\;\mathrm{Neative}}{\mathrm{True}\;\mathrm{Positive}\;+\mathrm{True}\;\mathrm{Neative}\;+\mathrm{False}\;\mathrm{Positive}\;+\mathrm{False}\;\mathrm{Neative}}$$

which indicates how often the model made an erroneous prediction;

(8) $$\mathrm{precision}=\frac{\mathrm{True}\;\mathrm{Positive}}{\mathrm{True}\;\mathrm{Positive}\;+\mathrm{False}\;\mathrm{Positive}}$$

which informs about the percentage of correctly predicted good joints;

(9) $$\mathrm{sensitivity}=\frac{\mathrm{True}\;\mathrm{Positive}}{\mathrm{True}\;\mathrm{Positive}\;+\mathrm{False}\;\mathrm{Negative}}$$

which corresponds to the percentage of ones that were correctly predicted by the model;

(10) $$\mathrm{specificity}=\frac{\mathrm{True}\;\mathrm{Negative}}{\mathrm{True}\;\mathrm{Neagtive}\;+\mathrm{False}\;\mathrm{Positive}}$$

which corresponds to the percentage of zeros that were correctly predicted by the model;

(11) $$F1\;\mathrm{score}=\frac{2\cdot \mathrm{precision}\;\cdot \;\mathrm{sensitivity}}{\mathrm{precision}\;+\;\mathrm{sensitivity}}$$

which is the harmonic mean of precision and sensitivity.

Then, the quality of the models regardless of the cut-off point was determined with the use of the ROC (Receiver Operating Characteristic) curve for all the obtained models, which is a continuous specificity and sensitivity curve for various cut-off points. The optimal cut-off point is the point closest to the point with coordinates (0,1), and therefore, the model for which the curve is closest to this point is the best. Finally, in order to compare all models, the AUC index was determined, being the area under the ROC curve, which is a measure of the goodness and validity of the model. The AUC value is in the range (0,1), and the dependency is as follows: the higher value—the better the model.

## 3. Results

### 3.1. Ultrasonic Testing Results

**Figure 4:**
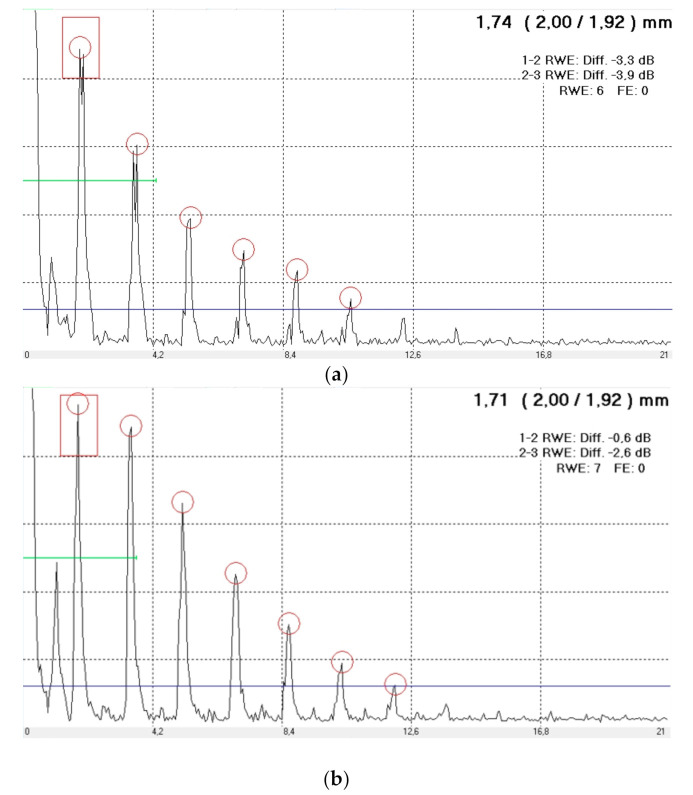

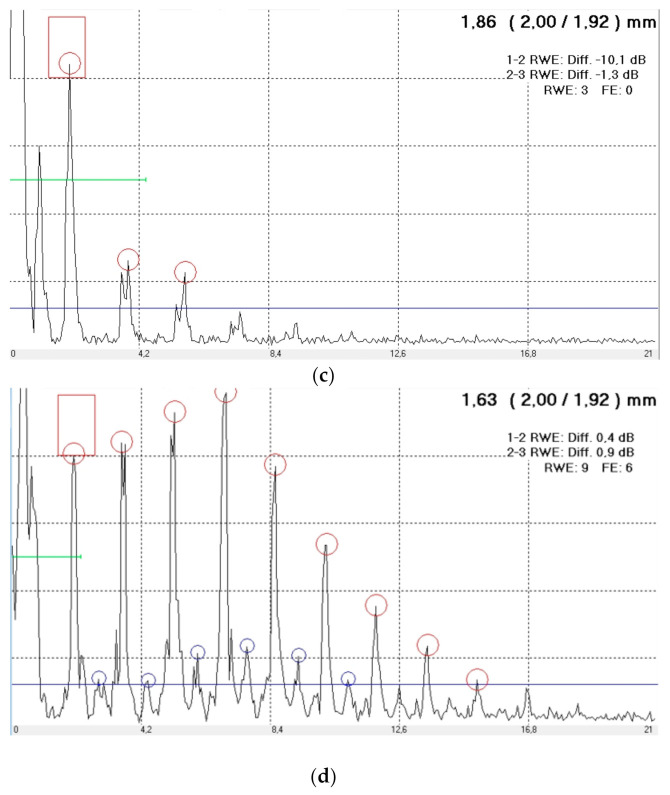
View of exemplary ultrasonic longitudinal wave pulses from the connection area: (**a**) high-quality connection, (**b**) sticking weld, (**c**) burnout connection due to high welding current, (**d**) too small weld nugget.

The results of ultrasonic tests for individual samples were recorded in the form of longitudinal ultrasonic waveforms diagram from the connection area. Exemplary impulses for different joint quality are shown in Figure 4. Based on the ultrasonic longitudinal wave impulse systems and parameters such as the number of reverse echoes, the number intermediate echoes, as well as the thickness of the connection in the place where the weld was formed, the connection quality was determined [28]. Exemplary results for selected welded connections are summarized in Table 2. Taking into account all the obtained connection results, the classification of the quality of the joint is as follows:

- High-quality connection;
- Connection with too small weld nugget;
- Sticking weld;
- Burnout connection.

Table 2 contains exemplary results (14 out of 75 results for mechanical tests and 14 out of 525 results for ultrasonic tests) of ultrasonic tests for samples welded using a different welding current. Only in the case of the lowest welding current (5.5 kA), intermediate echoes were observed, which indicate that the weld nugget is too small. For other welding currents, the number of repetitive echoes ranged from 5 to 6. After the welding process, the thickness of the sheets decreased with the increase in the welding current. It is related to faster plasticization of the material under the influence of current and the action of the electrode pressing force.

### 3.2. Strength of the Joint

**Figure 5:**
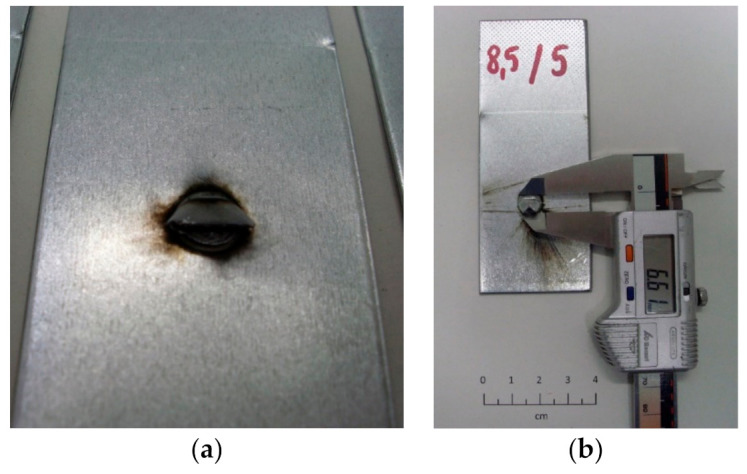
Weld nugget; (**a**) weld nugget view, (**b**) measurement of the diameter of the weld nugget with a caliper.

The shear test allowed determining the destructive force of the spot-welded joint. In the case of most joints, the sheet metal in the area of the welded joint was damaged. Only for the lowest welding current, damage at the connection point was noted, which is related to the too-small diameter of the weld nugget—the joining force of the sheets too low. Examples of the results of the breaking force of the connection are shown in Table 3. Additionally, the results of measurements of the diameter of the weld nugget after the shearing process are also included. The diameter was measured in two perpendicular directions, as shown in Figure 5. The lowest force of 3.0 kN was recorded for the welding current of 5.5 kA. As the welding current increases, the diameter of the spot weld nugget also increases. According to the standards of vehicle manufacturers, the diameter of the weld nugget for the tested sheet combination is 4 mm. All joints with a lower value (determined on the basis of ultrasonic tests or shear tests) are defective joints that do not meet the standards of the automotive industry.

The results of strength tests of spot-welded joints are lower than the values of the shear forces contained in [33]. However, in the process of sample preparation, not only the welding current was changed but also the time and the pressure of the electrodes. In addition, an ultrasonic head with a 5 mm transducer diameter was used, which proves a larger weld nugget and a more durable connection. However, a similar value of the shear forces and diameter of the weld nugget was obtained in [8,17,53]. These results confirm the correctness of the performance of spot-welded joints, the research of which is presented in the article.

## 4. Correlation of Mechanical Properties and Ultrasonic Parameters for a Spot-Welded Joint and Predictive Models of Quality of Joints

### 4.1. Basic Statistics and Correlation Analysis

During the research, 75 welded joints were tested (five samples for each welding current). Each sample was first examined with the ultrasound method, and then, the sheets were destroyed, while the force needed to break the connection and the diameter of the weld nugget was determined. On the basis of the diameter of the weld nugget determined from the shear test, as the average of two measurements made in perpendicular directions, 59 joints were classified as good joints, and 16 were classified as poor joints or no joints at all (then, the nugget diameter was recorded as 0 mm).

Basic descriptive statistics and confidence intervals at the level of 95% of the three tested parameters of the ultrasonic test for data classified as good joints and for data classified as weak joints are presented in the tables (Table 4 and Table 5).

**Figure 6:**
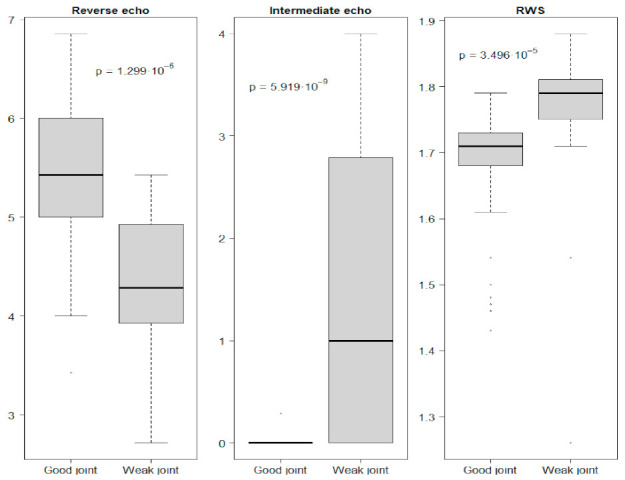
Parameter values obtained in the non-destructive testing.

**Figure 7:**
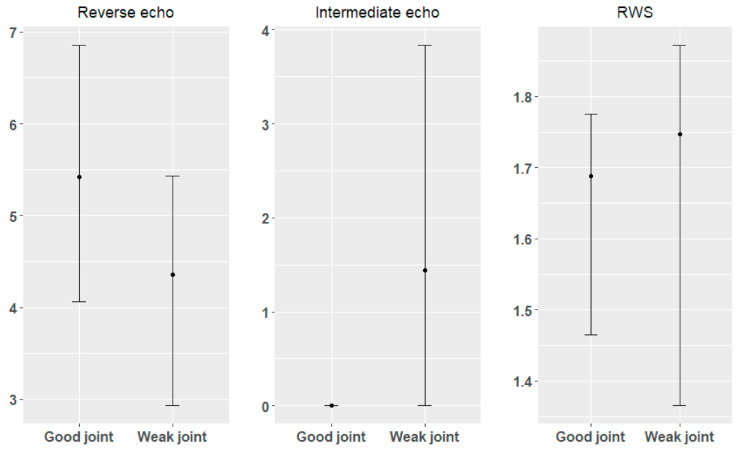
Confidence intervals of parameters from non-destructive testing for good and weak weld joints.

The number of reverse echoes measured for connections qualified as good was significantly higher than for connections qualified as weak, while the number of intermediate echoes (intermediate echoes practically did not occur in the case of a high-quality weld) and the RWS parameters were significantly lower for good connections (Figure 6). Confidence intervals determined at the significance level of 95% for good and weak joints (Figure 7) unfortunately mostly overlap, which makes it impossible to determine the reference intervals of these parameters for direct assessment of the quality of the weld. Lowering the materiality level will negatively affect the risk of a classification error.

**Figure 8:**
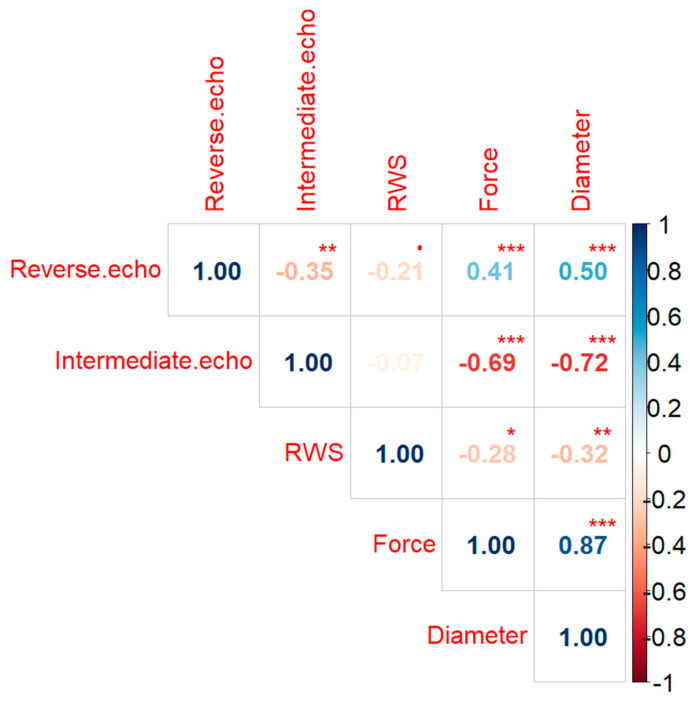
Correlation coefficients between the examined parameters, ‘.’ *p* = 0.1, ‘*’ *p* = 0.05, ‘**’ *p* = 0.01, ‘***’ *p* = 0.001.

Then, for the entire tested sample, the basic descriptive statistics of the three tested parameters of the ultrasonic test for the full data set were determined (Table 6), and the correlation matrix was prepared. Next, the significance of the determined correlation coefficients was checked (Figure 8). A negative correlation of 0.35 was found between the number of reverse echoes and the number intermediate echoes, but it was not significant enough to assume collinearities between the variables. However, there is a significant, strongly positive correlation between the number of reverse echoes and the force necessary to destroy the weld and for the diameter of the weld nugget. Then, a strong negative, statistically significant correlation between the number of echoes and the force as well as the diameter of weld nugget was observed. The negative correlation between the RWS parameter and the force and diameter for the tested data was definitely weaker and less statistically significant. The collinearity between the data determined by the ultrasound method and its occurrence in both constructed models was also checked using the variance inflation coefficient, and no collinearity was found.

### 4.2. Predictive Models

#### 4.2.1. Multiple Regression

Multiple regression analysis, where the predictors were the number of reverse echoes and the number of intermediate echoes and RWS, was performed independently for force and diameter. In addition, the analysis was performed twice—after the first attempt, two extreme values were rejected for each model based on Cook’s distance measure.

Finally, for the first model, the results of the analysis showed that the intermediate echo number and the RWS parameter were very strong negative predictors for the value of the force required to destroy the spot weld joint, while the number of reverse echoes was a strong positive predictor for the value of the force required to destroy the spot weld joint, and this model explains the 74.36% variation of the force needed required to destroy the spot weld joint. In the case of the second model, the number of intermediate echoes and the RWS parameter turned out to be very strong negative predictors of the weld nugget diameter. Additionally, the number of reverse echoes is a very strong positive predictor of the weld nugget diameter. This model explains the 74.93% variation in the weld nugget diameter (Table 7).

The models obtained on the basis of the data (Table 7) provide the formulas:

(12) Diameter=27.8174+0.7188⋅ReverseEcho−1.3924⋅IntemediateEcho−15.4138⋅RWS

(13) Force=14.03310+0.26602⋅ReverseEcho−0.39126⋅IntemediateEcho−6.34891⋅RWS

Equation (12) allows for the assumption that with the average RWS value for the full data set of 1.7 (Table 6) and in the absence of intermediate echoes, which for all samples classified as good connections did not occur (Table 4), the diameter of the weld nugget exceeds 4 mm (4.49 mm) for the number of reverse echoes of 4 and more. Assuming the maximum value of the RWS parameter in the full data set equal to 1.88 and assuming no intermediate echoes, the number of reverse echoes for which the weld nugget diameter exceeds 4 mm (4.59 mm) is 8 and more.

#### 4.2.2. Logistic Regression

Logistic regression analysis, where the predictors were the number of reverse echoes, the number of intermediate echoes, and RWS, was also performed for weld nugget diameter. The results of the analysis showed that the number of intermediate echoes and the RWS parameter turned out to be negative predictors of the weld nugget diameter (but only the RWS parameter is statistically significant), and the number of reverse echoes is a statistically significant positive predictor of the weld nugget diameter (Table 8).

The model obtained on the basis of the data (Table 8) provides the formula:

(14) $$\log\left(\frac{p}{1-p}\right)=104.542+2.684\cdot \mathrm{ReverseEcho}-20.445\cdot \mathrm{IntermediateEcho}-66.023\cdot \mathrm{RWS}$$

However, when analyzing the p-value, it can be concluded that both the logistic regression constant and the logistic regression coefficient for the intermediate echo do not have a statistically significant influence on the model result.

#### 4.2.3. Decision Tree

**Figure 9:**
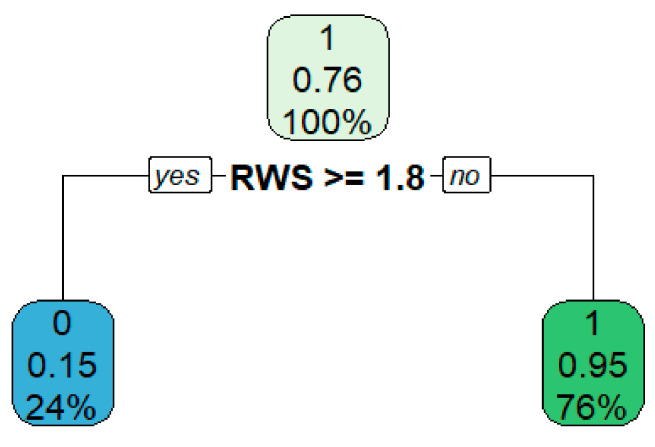
Decision tree for examined parameters.

The decision tree algorithm (where the predictors were the number of reverse echoes, the number of intermediate echoes, and RWS, and the dependent variable was the diameter of the weld nugget) has been implemented with the minimum number of observations in the node in order for a split to be attempted to 20, and with the minimum number of observations in the terminal node set to 7. As a result, the tree has only one decision rule, relative to the value of the RWS parameter set to 1.8 (Figure 9).

With a value of RWS parameter greater than or equal to 1.8, the sample is classified as a weak connection (there is only a 15% probability that a connection with this value of the RWS parameter has a good connection); otherwise, the connection is classified as good (with only 5% probability that connection with such value of the RWS parameter has a poor connection).

#### 4.2.4. Random Forest

The random forest algorithm (where the predictors were the number of reverse echoes, the number of intermediate echoes, and RWS, and the dependent variable was the diameter of the weld nugget) has been implemented with the number of trees set at 500 and with one variable tried at each split. The mean decrease in Gini coefficient, which is a measure of how each variable contributes to the homogeneity of the nodes and leaves in the resulting random forest obtained for the subsequent parameters was 5.204 for Reverse echo, 4.520 for Intermediate echo, and 7.778 for RWS parameter.

The higher the value of mean decrease Gini score, the higher the importance of the variable in the model; thus, in this model, the most important to the model is RWS parameter and the least important is the Intermediate echo.

### 4.3. Model Quality Verification

As part of the quality verification of the built models, they were tested on a set of testing data, and confusion matrices were built from the obtained predictions (Table 9).

Based on the confusion matrices, six parameters were determined for each model to verify their quality (Table 10).

Analyzing the accuracy, expressing how often the model correctly predicted the weld nugget quality, it can be seen that the logistic regression and the random forest show an identical 95% accuracy, while the accuracy of multiple regression and decision trees is 90%. Accordingly, the misclassification error rate, indicating how often the model made an erroneous prediction, is 5% for logistic regression and random forest, while for the other two models, it is 10%. Based on the precision parameter, informing about the percentage of correctly predicted ones, therefore in this case of a good weld nugget joint, both in the case of logistic regression and random forest, it is 100%—so each time a good joint was correctly identified by the constructed models. Multiple regression was characterized by a slightly lower precision of 94.12%, and the lowest precision of 89.74% was presented by the decision tree. In the case of the sensitivity test, corresponding to the percentage of ones that were correctly predicted by the model, the decision tree, which correctly classified all good connections in the test sample as good, was characterized by the highest 100% efficiency. The other three models had the same sensitivity of 94.12%. The highest specificity, corresponding to the percentage of zeros that were correctly predicted by the model, was characteristic for both logistic regression and the random forest—both models identified all weak joints as weak. Multiple regression correctly recognized 2/3 of the weak joints, while the decision tree recognized only 1/3. The last parameter, F1 score, being the harmonic mean of precision and sensitivity, averaging these values in a way, is the highest for logistic regression and random forest, amounting to 96.97% in both cases. The decision tree has a slightly lower value, i.e., 94.44%, and the lowest value, amounting to 94.12%, is a multiple regression.

**Figure 10:**
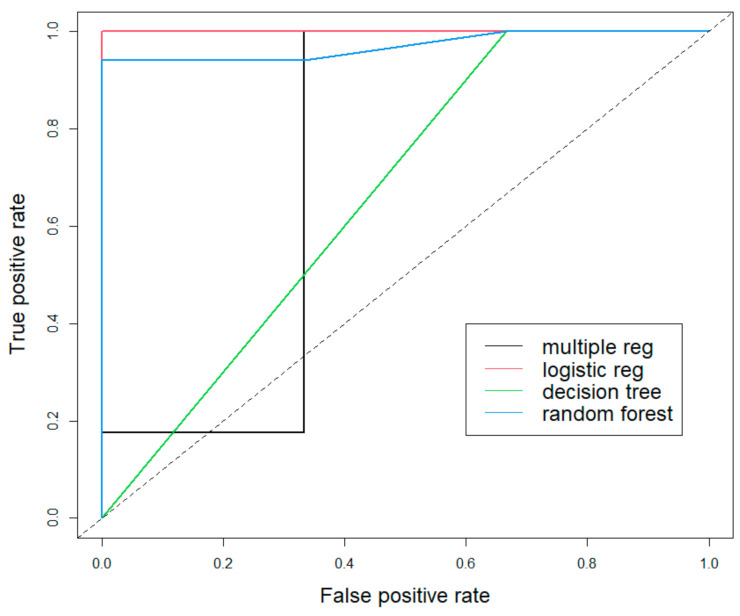
ROC curve for multiple regression model, logistic regression model, decision tree model, and random forest model.

Finally, the ROC curve was plotted (Figure 10), and the area under the curve (AUC) was calculated, obtaining values for the subsequent models, respectively: 0.725 for multiple regression, 1.000 for logistic regression, 0.667 for the decision tree, and 0.971 for the random forest.

Based on both the visual analysis (ROC curve) and the values of the calculated areas under the ROC curve (AUC), it can be seen that the logistic regression model (AUC equal to 1) is the most accurate, the second most accurate is the random forest (AUC equal to 0.971), then multiple regression and decision trees (with AUC values of 0.725 and 0.667 respectively).

The four predictive models for the weld nugget diameter—multiple regression, logistic regression, decision tree and random forest—were presented. Each of them was built on a set of training data to then be tested on a set of test data, and their quality was compared, using both quantitative parameters at the 50% cut-off point and the ROC curve and AUC values regardless of the cut-off point. The model built using logistic regression and the random forest at the 50% cut-off point showed identical accuracy (95%) and similar precision and specificity. The F1 score also indicates a higher accuracy of these models (Table 10). When examining the quality of the models using the ROC curve (Figure 10) and AUC values, the logistic regression model also shows the greatest validity, ahead of the random forest. In all tests, except for the sensitivity (which was the highest in the decision tree), both the multiple regression and the decision tree showed lower validity and were characterized by a greater error (Table 10).

When comparing the logistic regression model and the random forest model, the logistic regression model is supported by both the higher AUC value (equal to 1) and the fact that it is a model that, unlike the random forest, is easy to interpret.

### 4.4. Data Evaluation Using the Constructed Linear Regression Model

Additionally, performing multiple regression analysis allowed determining the ranges of the destructive force and the diameter of the weld nugget for the values of the parameters of the ultrasonic longitudinal wave. The synthetic results obtained by Equations (12) and (13) are summarized in Table 11.

## 5. Conclusions

Based on the research, the following statements can be made:

- The conducted analysis showed strong relationships between the obtained parameters of the non-destructive test (number of reverse echoes, number of intermediate echoes, RWS parameter) and the parameters of the destructive test (force and diameter of the nugget).
- The four models built in the work can be organized in terms of their accuracy and ease of interpretability as follows: logistic regression model, random forest, linear regression model, decision tree. Taking into account the very high accuracy of the first two of them (logistic regression and random forest) amounting to 95%, and very high, close to 100% values of precision, sensitivity, specificity, F1 index, and AUC values, they can be effectively used to classify the quality of weld nugget joints.
- For the selected combination of 0.8 and 1.2 mm thick sheets, it was found that the RWS parameter should be less than 1.8, which corresponds to a high-quality joint. In case of obtaining values equal to or higher than 1.8, the connection is characterized by too low strength.

Due to the number of tested samples (especially those where the connection was of poor quality), as well as the relatively large dispersion of data (Figure 6 and Figure 7), further research should focus on increasing the research sample and extending the parameters obtained from the ultrasonic test with results in the frequency domain. Extending the data with additional parameters from the non-destructive test may allow a more detailed explanation of the diameter of the weld nugget, which is the decisive parameter of the quality of the connection, improve the accuracy of the model, and minimize the error.

The results of research and analyses presented in the article are the first step toward developing a non-destructive system for estimating the strength of a spot-welded joint based on the ultrasonic wave parameters. By estimating the strength of spot-welded joints using the ultrasonic method, it will be possible to check the welded joints present in the area of a damage to the car body. This information will allow determining the extent of the repair. In the event of insufficient strength of welded joints weakened by the forces acting during a road accident, a decision to repair them can be made.

## Figures and Tables

**Table 1:** Confusion matrix.

		Predicted Condition	
		Good Joint	Weak Joint
Actual condition	Good joint	True Positive	False Negative
	Weak joint	False Positive	True Negative

**Table 2:** Selected results from ultrasonic measurements of spot-welded joints.

No.	Welding Current [kA]	Reverse Echos	Intermediate Echos	RWS
1	5.5	4	3	1.79
2	5.7	4	0	1.79
3	5.9	6	0	1.79
4	6.1	5	0	1.70
5	6.3	5	0	1.72
6	6.5	6	0	1.71
7	6.7	5	0	1.73
8	6.9	5	0	1.70
9	7.1	5	0	1.70
10	7.3	6	0	1.69
11	7.5	5	0	1.66
12	7.7	6	0	1.69
13	8.0	5	0	1.63
14	8.5	6	0	1.53

**Table 3:** Selected results from tear test and measurement of nugget diameter.

No.	Shear Test Force [kN]	Diameter of the Nugget [mm]
1	3.0	0
2	3.3	2
3	4.0	4.2
4	4.1	4.6
5	4.5	4.9
6	4.5	5.2
7	4.8	5.6
8	4.8	5.8
9	4.9	6.2
10	4.9	6.8
11	5.2	7.1
12	4.9	6.6
13	6.1	7.1
14	5.7	6.6

**Table 4:** Descriptive statistics of the tested parameters for data classified as good joints.

Parameter	Min.	1Q.	Median	Mean	Sd.	3Q.	Max.	Confidence Interval (2.5th–97.5th Percentile)
Reverse echo	3.429	5.000	5.429	5.424	0.736	6.000	6.857	4.064–6.857
Intermediate echo	0.000	0.000	0.000	0.005	0.037	0.000	0.286	0–0
RWS	1.430	1.680	1.710	1.688	0.080	1.730	1.790	1.465–1.776

**Table 5:** Descriptive statistics of the tested parameters for data classified as weak joints.

Parameter	Min.	1Q.	Median	Mean	Sd.	3Q.	Max.	Confidence Interval (2.5th–97.5th Percentile)
Reverse echo	2.714	3.964	4.286	4.357	0.767	4.893	5.429	2.929–5.428
Intermediate echo	0.000	0.000	1.000	1.438	1.536	2.679	4.000	0–3.839
RWS	1.260	1.765	1.790	1.748	0.151	1.805	1.880	1.365–1.873

**Table 6:** Descriptive statistics of the tested parameters for data classified as weak joints.

Parameter	Min.	1Q.	Median	Mean	Sd.	3Q.	Max.
Reverse echo	2.714	4.714	5.143	5.196	0.859	5.857	6.957
Intermediate echo	0.000	0.000	0.000	0.311	0.910	0.000	4.000
RWS	1.260	1.690	1.710	1.701	0.101	1.760	1.880

**Table 7:** The predictors of the multiple regression of force needed to break a weld and the nugget diameter (β-coefficient).

Parameter	Force	Diameter
(Intercept)	14.033 ***	27.817 ***
Reverse echo	0.266 **	0.719 ***
Intermediate echo	−0.391 ***	−1.392 ***
RWS	−6.349 ***	−15.414 ***
Multiple R^2^	0.758	0.764
Adjusted R^2^	0.744	0.749

‘**’ *p* = 0.01, ‘***’ *p* = 0.001.

**Table 8:** The predictors of the logistic regression of the nugget diameter (β-coefficient).

Parameter	Diameter
(Intercept)	104.542
Reverse echo	2.684 *
Intermediate echo	−20.445
RWS	−66.023 *

‘*’ *p* = 0.05.

**Table 9:** Confusion matrix for multiple linear regression, logistic regression, decision tree, and random forest.

Multiple Regression				Logistic Regression		Decision Tree		Random Forest	
		Predicted Condition		Predicted Condition		Predicted Condition		Predicted Condition	
		Good Joint	Weak Joint	Good Joint	Weak Joint	Good Joint	Weak Joint	Good Joint	Weak Joint
Actual condition	Good joint	16	1	16	16	17	0	16	1
	Weak joint	1	2	0	3	2	1	0	3

**Table 10:** Accuracy, MER, precision, sensitivity, specificity, and F1 score obtained for each model on the testing data.

	Multiple Regression	Logistic Regression	Decision Tree	Random Forest
Accuracy	0.900	0.950	0.900	0.950
MER	0.100	0.050	0.100	0.050
Precision	0.941	1.000	0.895	1.000
Sensitivity	0.941	0.941	1.000	0.941
Specificity	0.667	1.000	0.333	1.000
F1 score	0.941	0.970	0.944	0.970

**Table 11:** Relationships between the ultrasonic wave parameters and the mechanical strength of the connection obtained by multiple regression model.

Reverse Echo	Intermediate Echo	RWS	Force kN	Diameter mm
<3	>0	Min–Q1	2.71–6.17	0–8.44
		Q1–Q3	2.08–3.51	0–1.97
		Q3–Max	1.31–2.88	0–0.43
<3	0	Min–Q1	3.49–6.57	2.23–9.82
		Q1–Q3	2.86–3.90	0.69–3.36
		Q3–Max	2.26–1.82	0–1.82
3	0	Min–Q1	2.90–3.53	1.00–2.54
		Q1–Q3	3.66.4.16	2.85–4.08
		Q3–Max	4.29–6.83	4.39–10.55
4	0	Min–Q1	3.16–3.80	1.71–3.26
		Q1–Q3	3.92–4.43	3.56–4.80
		Q3–Max	4.56–7.10	5.11–11.27
5	0	Min–Q1	3.43–4.06	2.43–3.97
		Q1–Q3	4.19–4.70	4.28–5.52
		Q3–Max	4.82–7.36	5.82–11.99
6	0	Min–Q1	3.69–4.33	3.15–4.69
		Q1–Q3	4.46–4.96	5.00–6.24
		Q3–Max	5.09–7.63	6.54–12.71

## Data Availability

The data presented in this study are available on request from the corresponding author.
